# Changes in health and primary health care use of Moroccan and Turkish migrants between 2001 and 2005: a longitudinal study

**DOI:** 10.1186/1471-2458-8-40

**Published:** 2008-01-29

**Authors:** Majda Lamkaddem, Peter M Spreeuwenberg, Walter L Devillé, Marleen Foets, Peter P Groenewegen

**Affiliations:** 1 NIVEL (Netherlands Institute for health services Research), Utrecht, The Netherlands; 2 IBMG (Institute of Health Policy and Management), Erasmus University, Rotterdam, The Netherlands; 3 Utrecht University, dep. of Sociology, dep. of Human Geography, The Netherlands

## Abstract

**Background:**

Social environment and health status are related, and changes affecting social relations may also affect the general health state of a group. During the past few years, several events have affected the relationships between Muslim immigrants and the non-immigrant population in many countries. This study investigates whether the health status of the Moroccan and Turkish immigrants in the Netherlands has changed in four years, whether changes in health status have had any influence on primary health care use, and which socio-demographic factors might explain this relationship.

**Methods:**

A cohort of 108 Turkish and 102 Moroccan respondents were interviewed in 2001 and in 2005. The questionnaire included the SF-36 and the GP contact frequency (in the past two months). Interviews were conducted in the language preferred by the respondents. Data were analysed using multivariate linear regression.

**Results:**

The mental health of the Moroccan group improved between 2001 and 2005. Physical health remained unchanged for both groups. The number of GP contacts decreased with half a contact/2 months among the Turkish group. Significant predictors of physical health change were: age, educational level. For mental health change, these were: ethnicity, age, civil status, work situation in 2001, change in work situation. For change in GP contacts: ethnicity, age and change in mental and physical health.

**Conclusion:**

Changes in health status concerned the mental health component. Changes in health status were paired with changes in health care utilization. Among the Turkish group, an unexpected decrease in GP contacts was noticed, whilst showing a generally unchanged health status. Further research taking perceived quality of care into account might help shedding some light on this outcome.

## Background

The Netherlands is one of Europe's traditional immigration countries, and is therefore quite multicultural. In 2005, almost 20% of the population were of foreign origin [1]. First and second generation immigrants of Moroccan and Turkish origin account for more than one fifth of the total number of foreigners in the country. These two groups are together with the Surinamese and the Antillean people the four largest minority groups in the Netherlands.

The Turkish and Moroccan immigration in the Netherlands dates back from the sixties, when Dutch industries started to recruit abroad in order to attract extra labour forces. This plan was targeted at different countries of the Mediterranean, and was a joint effort of several Western European nations. As a result, between the end of World War II and the mid-seventies, guest workers came to the Netherlands and other North European countries, departing from several countries of the Mediterranean, including Spain, Italy, Morocco and Turkey. What was first meant to be a temporary migration turned into a permanent stay, as many immigrants settled in the Netherlands and had later their families come over for reunification [2].

Since the 2002 electoral victory of Lijst Pim Fortuyn (LPF), led by a right-wing populist, it became clear that the tensions between the Dutch and what is commonly defined as Muslim groups were very present, and that the agreement on a multicultural society no longer existed. The murder of the controversial film director and columnist Theo van Gogh some years later by a Muslim extremist was viewed by the public as yet another sign that the "integration" of the Muslim groups had not been successful [3].

Regardless of the interpretation of these and other events that characterized the political scene of the Netherlands in the past five years, it is interesting to find out whether these changes in social climate affected the perceived health of the groups involved.

According to Andersen [4], the environment in which health is being defined and measured influences health outcomes. In his latest model, Andersen related health outcomes to the environment, implying and recommending the use of longitudinal study designs in order to implement it. This approach allows health to be conceived as a variable concept, subject to change as time passes and societal situations vary.

Therefore, we decided to take a longitudinal approach in order to examine possible changes in the health status and GP health care use of Turkish and Moroccan people in the Netherlands. This kind of approach is new in research linking mental and physical health and health care use of migrant groups in the Netherlands, where a strong gate keeping system exists between primary and specialist health care. Generally, legal migrant groups have the same access to primary health care as the Dutch. The health system is further characterized by a mix of fee-for-service and capitation, which aims at equal access to primary health care for all.

Within the conceptualisation of Andersen, two questions – concerning the relationships between needs and health behaviour, and their evolution over time – are the focus of this paper. The first question concerns the evolution of health care need over time. Does self-reported health status (mental and physical) vary over time, taking socio-demographic characteristics into account, and is there a difference between ethnic groups in this regard? The second question concerns the evolution of the relationship between self-reported health (need) and the frequency of GP contacts (health care utilization). Does this relationship vary over time, and, if this is the case, is the difference between 2001 and 2005 similar in importance and direction for both ethnic groups?

## Methods

### Study design

For this research, we have chosen a longitudinal study design. The data issue from two similar surveys conducted around 2001 (T1) and 2005 (T2) among a sample of 220 respondents.

The first interview round took place within the Second Dutch National Survey of General Practice (DNSGP-2) [5]. This study was conducted within a geographically representative sample of 104 general practices across the country. At this point, it is important to note that in the Netherlands, patients are registered at one general practice only. Their medical records are also centralised at that practice. A random sample of these patients was asked to take part in a survey about their health and health behaviour. This survey was also conducted among the four main foreign groups in the Netherlands. Among these, 797 people of Turkish and Moroccan origin aged 18 and over were interviewed.

### Response rate

Those respondents were asked for their consent to take part in a follow-up interview, and 573 (72%) agreed. It was not easy to find the respondents again after four years. Many had moved and were no longer registered at the same general practice. We used the general practices in order to contact the potential respondents. Most practices agreed to collaborate and to send a joint information letter about the second survey. Within one week, interviewers were instructed to call respondents of whom a telephone number was registered first and make an appointment for the interview. In a few practices the GPs did not agree to collaborate; in these cases we have sent a letter directly to the respondents, following the same action points as mentioned above. Interviewers visited people who could not be contacted by telephone. A reminder was sent to people who did not answer the first letter; we were not sure about their addresses. We asked them to contact us. Eventually, 118 Turkish and 102 Moroccan respondents took part in the second survey (38.4% of the respondents who agreed at T1).

Of the 354 that did not take part in T2 while they initially agreed, 51 (8.9% of the 573 potential respondents) refused, and 292 (50.9%) could not be reached (either they had moved, or they were not at home). Eleven potential respondents (1.9%) were initially excluded from this survey because they were living at places that could hardly be reached by public transport.

### Interviews and measuring instruments

Interviews (T1 and T2) were conducted in Dutch, Moroccan, Arabic or Turkish, according to the respondent's choice. The ethnic background of the interviewers matched that of the respondents. The interview was based on a structured questionnaire, containing, among other things, translations of the Short Form Health survey-36 (SF-36, or Rand-36) as measuring instrument for health status (Dutch version validated [6]). The translations of the SF-36 in Turkish and Moroccan-Arabic were in the process of being validated at the time of the study, by Hoopman et al.[7]. Several items were collected on demographic and socio-economic characteristics. The work situation was also recorded, and further dichotomised in working (employed) and non-working respondents (including students). The change in working situation was computed as the difference between both measurement moments in employment (unemployed to employed = "better", employed to unemployed = "worse", the other two possibilities are coded as "no change"). Change in civil status was also computed in the same way, resulting in 3 categories ("from having no partner to having a partner", "no change", "from having a partner to having no partner"). Respondents were also asked about their health care use, specifically the contact frequency with the GP in the past two months.

The SF-36 consists of eight dimensions, each measuring one aspect of health status (i.e. physical functioning, social functioning, role limitations due to physical problems, role limitations due to emotional problems, mental health, pain, vitality and general health perception). For the purpose of this study, these eight scales have been summarized into two components: the Physical Component Summary Scale (PCS) and the Mental Component Summary Scale (MCS), using the scoring algorithms as presented by Ware et al. [8].

In constructing these two summary measures, the eight scales of the SF-36 are standardized using means and standard deviations from the general US population [8]. A study by Aaronson et al. [9] showed that the US figures only slightly differ from the general Dutch population. Therefore, and in order to facilitate international comparison, we have chosen to use US figures for standardizing the scores. Scores below 50 on one or both scales mean a worse health than that of the general population, and scores above 50 indicate a better health than that of the general population. Both summary scales were computed for T1 and T2.

### Missing data

Missing data for items of the SF-36 at T1 and T2 were replaced using a model-based single imputation for each item. The data set with replacement of the missing values was only used for power purposes in the multivariate analyses. Other analyses show results without replacement of missing data.

### Analyses and outcome measures

For the bivariate analyses, paired-sample t-tests were used to test differences between the two time-points. The multivariate models were built using linear regressions. The three dependent variables were: change in mental health status, change in physical health status and change in number of GP contacts in the past two months. These outcome variables were delta variables, calculated X = xT2 - xT1, where a positive outcome indicates an increase, and a negative outcome a decrease. In all analyses, changes have been adjusted for the baseline value (value on T1), as larger increases are more likely to happen for those with a lower value on T1 [10]. Furthermore, socio-demographic characteristics taken in the models are measured on T2, except for change variables, measured on both time-points as X = xT2 - xT1. The analyses were performed using SPSS 11.5.

The study was carried out according to Dutch legislation on privacy. The privacy regulation of the study was approved by the Dutch Data Protection Authority. According to Dutch legislation, obtaining informed consent is not obligatory for observational studies.

## Results

### Non response analysis

Most socio-demographic characteristics do not differ significantly between respondents on the first wave only and respondents on both waves. One difference is that respondents on both waves were slightly younger than respondents on T1 only (OR = 0.980, p = 0.028). With added information about mental and physical self-reported health status to the model, respondents on T2 still do not differ significantly from their sample frame on these health and socio-demographic characteristics, except from being younger, and reporting a slightly worse mental health, although just not significant (p = 0.073). (Table 1).

**Table 1:** Odds of taking part in T2 according to socio-demographic characteristics and physical and mental self-reported health at T1 (N = 670)^a^

	Model 1		Model 2	
	Odds Ratio (95% CI)	p-value	Odds Ratio (95% CI)	p-value

*Gender (ref: male)*				
Female	0.991 (0.668; 1.470)	0.962	0.946 (0.633; 1.413)	0.785
*Age (years)*	0.980 (0.962; 0.998)	0.028	0.979 (0.961; 0.998)	0.029
*Ethnicity (ref: Turkey)*				
Morocco	0.895 (0.633; 1.267)	0.532	0.920 (0.648; 1.305)	0.639
*Education *(ref:Vocational/University)				
None/primary	1.417 (0.634; 3.166)	0.396	1.381 (0.616; 3.095)	0.433
Secondary	1.282 (0.601; 2.734)	0.520	1.250 (0.585; 2.670)	0.565
*Born in the Netherlands *(ref:no)				
Yes	0.900 (0.507; 1.595)	0.717	0.920 (0.518; 1.634)	0.776
*Civil status *(ref: widower)				
Unmarried	0.436 (0.119; 1.596)	0.210	0.460 (0.124; 1.699)	0.244
Married/partner	0.698 (0.226; 2.152)	0.531	0.732 (0.235; 2.278)	0.590
Divorced	0.636 (0.172; 2.355)	0.498	0.598 (0.160; 2.233)	0.444
*Work situation *(ref: unemployed)				
Employed	1.152 (0.781; 1.699)	0.477	1.198 (0.807; 1.779)	0.371

*Mental health*			0.985 (0.969; 1.001)	0.073
*Physical health*			1.002 (0.982;1.022)	0.871

^a ^SF-36 summary scales, standardized to the general US population (see methods), without replacement of missing values

### Socio-demographic characteristics and health status of the study population

On average, the Turkish and the Moroccan groups did not differ in age (respectively 38 and 40 years of average, p = 0,256), and the first generation of both groups was not showing a different average length of stay (the Turkish first generation had been in the Netherlands for 22 years, the Moroccan one for 21 years, p = 0,182). Concerning the proportion of first and second generation migrants, we see that 21.6% of the Moroccan group are born in the Netherlands, against 12,7% of the Turkish group (p = 0,081). Finally, the proportion of male and female respondents did not differ between both groups (p = 0,263).

For the eight presented SF-36 health dimensions, the Turkish and the Moroccan did not show any significant differences on T1. However, on T2, the Moroccan group showed better scores on social functioning (p = 0.003) and mental health (p = 0.000) than the Turkish (Table 2). Within ethnic groups, the only significant difference between both time-points concerned the mental health of the Moroccan group that improved between T1 and T2. On social functioning, this was almost significant (p = 0.068). The Turkish group did not show any significant difference between measurement times. (Table 2).

**Table 2:** SF-36 health dimensions scores (unstandardized)^a^

	*Moroccan (n = 102)*		*Turkish (n = 118)*	
	***T1 Mean score (SD)***	***T2 Mean score (SD)***	***T1 Mean score (SD)***	***T2 Mean score (SD)***

*Physical functioning*	84.9 (21.3)	84.5 (21.9)	82.8 (21.4)	81.8 (25.4)
*Social functioning*	77.4 (25.0)	82.4 (20.4)	75.6 (25.9)	73.8 (22.3)
*Role limitation physical*	75.0 (39.2)	74.5 (37.7)	73.9 (40.7)	71.1 (41.4)
*Role limitation emotional*	78.1 (38.5)	85.0 (32.5)	79.6 (37.1)	74.3 (42.2)
*Mental health*	**64.5 (21.8)**	**72.7 (14.6)**	63.4 (22.7)	62.5 (19.8)
*Vitality*	53.4 (19.1)	56.8 (15.1)	54.7 (22.9)	55.1 (19.4)
*Pain*	77.3 (29.6)	81.9 (23.5)	70.7 (31.7)	75.5 (29.6)
*General health*	57.9 (24.3)	60.2	59.4 (22.6)	56.8 (21.9)

^a ^Bold letter type indicates a significant difference between time points at p = 0.05

### Changes in health status and GP health care use

**Figure 1:**
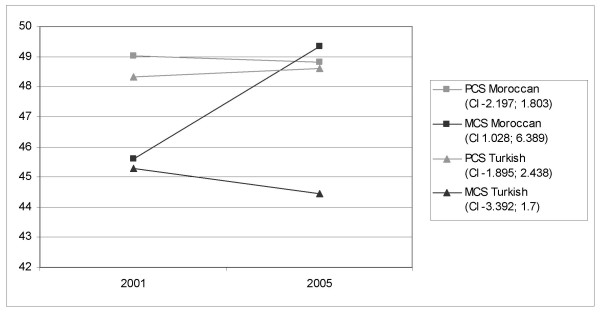
Changes in (self-reported) MCS and PCS between 2001 and 2005.

When summarizing the SF-36 items into two components (MCS and PCS), the Moroccan group shows a significant improvement in mental health, with a mean difference of 3,709 (95% CI 1,028;6,389) (p = 0.007). Other changes in time are not significant (p > 0.1) (Figure 1).

**Figure 2:**
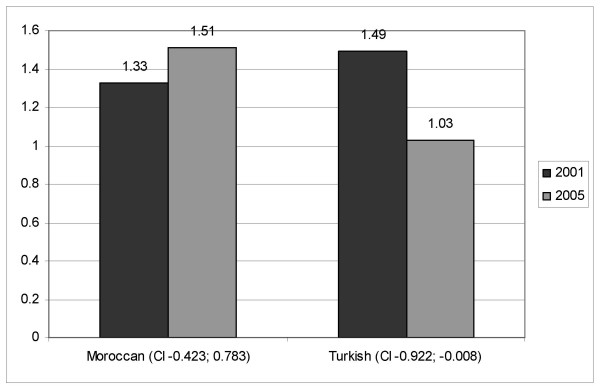
Changes in (self-reported) number of GP contacts (past 2 months) between 2001 and 2005.

On the reported use of GP care (Fig. 2), the Turkish group reports significantly less contacts in 2005 than in 2001 (p = 0.046). The difference accounts for half a contact/2 months. The Moroccan group shows an unchanged average (p = 0.555).

### Predicting changes in self-reported health and health care use

Table 3 displays the linear regression coefficients for change in physical health status, change in mental health and change in GP contacts. Concerning physical health change, age is associated with a decrease in physical health, and a higher educational level is associated with an improvement in physical health. Ethnicity did not predict changes in physical health between 2001 and 2005.

**Table 3:** Predictors of physical health change, mental health change and change in GP contacts (past two months) between 2001 and 2005: linear regression coefficients^a^

	PCS change	MCS change	GP contacts change
	Regression coefficients (95% CI)	Regression coefficients (95% CI)	Regression coefficients (95% CI)

	Adj. R^2 ^= 0.389	Adj. R^2 ^= 0.619	Adj. R^2 ^= 0.584

*PCS T1*	**-0.730 (-0.862;-0.598)**		
*MCS T1*		**-0.945(-1.054;-0.835)**	
*Number of contacts on T1*			**-0.850 (-0.976;-0.724)**
*Change in PCS*			**-1.462 (-2.337;-0.587)**
*Change in MCS*			**-0.675 (-1.102;-0.247)**
*Male (ref: female)*	1.296 (-1.604;4.197)	0.093 (-2.816;3.003)	-0.191 (-0.838;0.455)
*Moroccan (ref: Turkish)*	0.995 (-1.572;3.562)	**3.877 (1.343; 6.412)**	**0.589 (0.029;1.150)**
*Age (years)*	**-0.223 (-0.356;-0.091)**	**0.136 (0.008; 0.264)**	**0.045 (0.016;0.074)**
*Civil status*			
married/registered partner	ref	ref	ref
Not married	-1.805 (-6.477;2.867)	2.872 (-1.768;7.513)	-0.103 (-1.142; 0.936)
divorced	-0.232 (-5.790;5.326)	**-7.463 (-13.004;-1.923)**	0.009 (-1.242; 1.259)
*Work situation*			
unemployed	ref	ref	ref
employed	2.240 (-1.284;5.764)	**4.621 (1.106;8.136)**	-0.191 (-0.966;0.584)
*Educational level*	**3.157 (0.697;5.616)**	1.106 (-1.333;3.545)	-0.438 (-0.982; 0.106)
*Civil status change*			
No change	ref	ref	ref
From partner to no partner	3.110 (-2.665;8.885)	0.331 (-5.399;6.061)	-0.156 (-1.428;1.116)
From no partner to partner	-3.962 (-8.669;0.745)	-1.207 (-5.932;3.519)	-0.491 (-1.513;0.530)
*Work situation*			
No change	ref	ref	ref
From employed to unemployed	-1.406 (-5.238;2.425)	3.529 (-7.334; 0.275)	0.043 (-0.787;0.873)
From unemployed to employed	1.427 (-2.680;5.535)	**5.638 (1.563; 9.714)**	-0.124 (-1.063;0.815)

^a ^Bold letter type indicates a significant association at p = 0.05

On mental health change, being Moroccan is positively associated with an improvement in mental health, while being divorced compared to being married/having a partner is associated with a decrease in mental health. Being employed in 2001 predicts an improvement in mental health status, and a further positive change in work situation between 2001 and 2005 is also associated with an improvement in mental health. For this model, age is associated with an improvement in mental health.

Concerning the change in GP contacts in the past two months, improvement in mental and physical health both relate to a decrease in number of GP contacts. Ethnicity also plays a role in explaining the change in GP contacts: the Moroccan group shows a larger increase in GP contacts than the Turkish group. Likewise, being older is positively associated with a larger increase of GP contacts. Other associations with other socio-demographic variables are not significant.

## Discussion

The answer to our first research question on whether the perceived health status of Turkish and Moroccan (children of) immigrants has changed over time is that the mental health of the Moroccan group improved between 2001 and 2005, making this group significantly healthier than the Turkish group in 2005. Physical health status of both groups did not change in time.

The second focus question of this paper was whether changes in health status were paired with a change in health care utilization. In our results, the Turkish group shows a significant decrease in GP contacts during the past two months, while the Moroccan group does not show any change. In the multivariate model, changes in GP contacts were significantly explained by changes in mental and physical health.

### Study limitations

This study is based on self-report. The chosen measuring instrument might not be valid in a cross-cultural perspective, although the chosen 36-items health questionnaire might be more reliable than the often used one-item measurement [11]. Likewise, self-report in the number of contacts with the GP is also subject to possible bias (for instance recalling incorrectly the exact number of consultations with the GP in the past two months), and would need to be compared with information in medical records in order to assess its validity. However, a previous study indicates that self-report provides a valid estimation of ethnic differences in health care use [12].

Some kind of selectivity might have taken place in the response, as only 27.6% of the respondents on the first wave also took part to the second wave. However analysis of both groups for socio-demographic characteristics showed no significant difference between them, except for a slightly younger age.

Finally, small sample sizes carry the risk of not revealing some relevant statistical associations. This is often the case in research among groups hard to contact, such as ethnic minorities [13].

### Changes in health status

In 2001, both groups scored under the population norm on their physical and mental health status. As explained in the methods section, the U.S. norm has been proven comparable to that of the general Dutch population. In 2005, the Moroccan group reaches the norm on mental health status, as opposed to the Turkish group that remains under that norm. Obviously, comparison with the general population is also time-dependent, as the standardization is based on figures dating back from the 1990s. As our data show, self-reported health varies in time, and it is likely that this is also the case for the general population. It is therefore impossible to draw definite conclusions for the comparison of health status of both groups with the general population on the two different time-points. Standardization occurred mainly for scale-construction purposes in this paper (see Ware et al. [8]).

In the multivariate models, some differences between both health indicators changes need to be highlighted. One of them is the coincidence of change in work situation and mental health change. This relationship does not exist for physical health change, where a positive or negative change of the work situation does not seem to play a part. Another interesting difference is the propensity of the Moroccan group, compared to the Turkish one, to see improvement of their mental health status in time, while no difference between both groups is shown on physical health status change. Likewise, civil status seems to play a role in change in mental health, with divorced respondents more likely to show a decrease in mental health. This is not the case for physical health, where, in turn, educational level seems to be more relevant for explaining changes (people with a higher educational level are more likely to have experienced an improvement in physical health in the past four years).

The main difference regarding health status on both time points concerns therefore the mental component. The Turkish group has a significantly worse mental health status in 2005 than the Moroccan group. It can therefore be argued that part of the explanation could be found in cultural differences. The measuring instrument has been translated and is in the process of being validated within each group. However, this does not allow any conclusion on cross-cultural comparability of its outcomes. In a recent paper, Agyemang et al. [11] showed that the one-item self-reported health status was not valid in a cross-cultural perspective, arguing that different groups rated their health differently while showing comparable morbidity figures. The cultural component could also play a role in using the 36 items of our instrument, although factual information rather than perception of general health is the starting point of this component. However, other differences could play a role in explaining the difference in mental health. Several media, opinion polls and research findings all pinpoint the Turkish group as less satisfied with the social climate in the Netherlands. A recently published research report of the Social and Cultural planning Office of the Netherlands [13] shows that the Turkish group stands out as having the most negative views on matters such as acceptation of the own group by the Dutch, and the hospitality of the Dutch. Likewise, the Turkish group has a more negative view on the Dutch than the Moroccan group does. In line with these results, the media have sometimes emphasized the fact that the wish for return migration is stronger among Turkish people than among Moroccan people [14,15].

An interesting next step in the presented analyses would be to take several cultural and discrimination indicators, and examine how these explain the relatively different mental health status of our sample. As earlier mentioned, multiculturalism is no longer agreed upon in the Netherlands. The way ethnic minorities perceive this fact can influence their (mental) health status. Moreover, differences between groups might exist, as to how cultural tensions are perceived, and this might, in turn, lead to differences in (mental) health status.

### Changes in health care use

When we examine variables that can explain changes in GP health care use, we can see that changes in mental and physical status largely explain the variation in the change (adj. R^2 ^= 0.535), with an improvement in health status being associated with a decrease in health care use. When we add the socio-demographic characteristics to the model, age and ethnicity turn out to be the only predictors of change in health care use, with the Moroccan group and the elderly showing a larger propensity to increase their health care use.

Data on self-reported health care use among Dutch patients are not available for 2005, but registration data show that the yearly average of GP contacts among Dutch patients increased by 8,4% between 2001 and 2005, which is less than the increase reported by Moroccan respondents [16].

A step we consider interesting at this point would be to look at the perception of the quality of GP care and its change over time and to examine if, and how far it could explain differences between both groups in changes in GP consultation. We could hypothesize that patients who are less satisfied about the quality of GP care would call less often upon their GP, especially if their physical health (vs. mental health) is not at stake. Likewise, changes in use of alternative health care, such as ethnic medicine or health care in the country of origin could possibly play a role in such a development. Our data show that Turkish and Moroccan groups do not differ significantly in their use of health care in the country of origin, but that they use health care in the country of origin considerably, i.e. 12, 7% of the Moroccan and 16, 9% of the Turkish made use of some kind of health care in their country of origin in the past year, 2001. Unfortunately, we do not have this information for 2005, which makes investigating changes impossible. This forms another point on the research agenda, in addition to the possible influence of perceived discrimination.

## Conclusion

This study has shown that changes in perceived health status between 2001 and 2005 among Turkish and Moroccan (children of) immigrants concerned the mental health dimension. The mental health status of the Moroccan group improved between 2001 and 2005, making this group significantly healthier than the Turkish group in 2005. Physical health status of both groups did not change over time. Multivariate models examined factors of change in mental and physical health, where both socio-demographic and health variables were significant predictors, as well as a change in work situation for mental health.

Secondly, the analyses showed that changes in health status were paired with a change in health care utilization. Our results show that, firstly, the Turkish group shows a significant decrease in contacts with the GP in the past two months, while the Moroccan group does not show any change. Secondly, when examining which variables can explain changes in GP health care use, we can see that improvements in mental and physical status broadly explain decreases in GP contacts. When we add the socio-demographic characteristics to the model, we notice that age and ethnicity are the only predictors of change in health care use, with the Moroccan group and the elderly showing a larger propensity to increase their health care use.

One point standing out of these results is the unexpected decrease in GP contacts among the Turkish group, while showing a generally unchanged health status. Further research taking perceived quality of care, use of alternative health care and health care in the country of origin into account, might help shedding some light on this outcome.

## Competing interests

The author(s) declare that they have no competing interests.

## Authors' contributions

ML carried out the research, performed the statistical analysis and drafted the manuscript. PMS gave methodological and statistical guidance. WLD participated in the study design, supervised the data collection and gave comments on the manuscript. MF conceived the study and commented the manuscript. PPG gave methodological guidance and commented the manuscript. All authors read and approved the final manuscript

## Pre-publication history

The pre-publication history for this paper can be accessed here:


